# The Impact of Exercise Training Intensity on Physiological Adaptations and Insulin Resistance in Women with Abdominal Obesity

**DOI:** 10.3390/healthcare10122533

**Published:** 2022-12-14

**Authors:** Nourhen Mezghani, Achraf Ammar, Omar Boukhris, Rihab Abid, Atyh Hadadi, Turki Mohsen Alzahrani, Omar Trabelsi, Mohamed Ali Boujelbane, Liwa Masmoudi, Ibrahim Ouergui, Kamel Jamoussi, Mouna Mnif, Hafedh Mejdoub, Piotr Zmijewski, Jordan M. Glenn, Khaled Trabelsi, Hamdi Chtourou

**Affiliations:** 1 Department of Sport Sciences, College of Education, Taif University, P.O. Box 11099, Taif 21944, Saudi Arabia; 2 Department of Training and Movement Science, Institute of Sport Science, Johannes Gutenberg-University Mainz, 55099 Mainz, Germany; 3 Interdisciplinary Laboratory in Neurosciences, Physiology and Psychology: Physical Activity, Health and Learning (LINP2), UFR STAPS, UPL, Paris Nanterre University, 39200 Nanterre, France; 4 High Institute of Sport and Physical Education, University of Sfax, Sfax 3038, Tunisia; 5 Physical Activity, Sport, and Health, UR18JS01, National Observatory of Sport, Tunis 1003, Tunisia; 6 Sport and Exercise Science, School of Allied Health, Human Services and Sport, La Trobe University, Melbourne 3086, Australia; 7 Research Laboratory, Education, Motricity, Sport and Health (EM2S), LR15JS01, High Institute of Sport and Physical Education, University of Sfax, Sfax 3038, Tunisia; 8 High Institute of Sport and Physical Education of Kef, University of Jendouba, El Kef 7100, Tunisia; 9 Laboratory of Biochemistry, CHU Hedi Chaker, University of Sfax, Sfax 3000, Tunisia; 10 Department of Endocrinology, Hedi Chaker University Hospital of Sfax, Sfax 3038, Tunisia; 11 Laboratory of Plant Biotechnology, Sfax Faculty of Sciences, BP 1171, University of Sfax, Sfax 3038, Tunisia; 12 Jozef Pilsudski University of Physical Education in Warsaw, 00-809 Warsaw, Poland; 13 Department of Health, Exercise Science Research Center Human Performance and Recreation, University of Arkansas, Fayetteville, AR 72701, USA

**Keywords:** abdominal obesity, training, intensity, insulin resistance

## Abstract

Abdominal obesity has emerged globally as a major public health issue due to its high prevalence and morbidity. The benefits of physical exercise among the obese population are well documented. However, the optimal exercise intensity for reducing body fat and preventing insulin resistance and metabolic disorders is still under debate. This study aimed to examine the effects of three different intensities of combined endurance and strength training programs on anthropometric variables, physiological and muscular adaptations, and insulin sensitivity. Forty-three obese young women (age 26.4 ± 4.7 years, BMI 33.1 ± 2.5 kg/m^2^) were randomly assigned to one of four groups: a control group (G0), a moderate-intensity training group (G50, exercising brisk walking at 50% heart rate reserve HRR), a high-intensity training group (G75, exercise jogging at 75% HRR), and an alternated-intensity training group (G50/75, exercise brisk-walking/jogging at 50–75% HRR) with additional strength training once a week for each group. Body composition, waist circumference (WC), fasting blood glucose, insulin sensitivity and resistance (Homa-IR), resting heart rate (RHR), 6-min walk distance (6MWD), 1-repetition maximum (1-RM), and time to exhaustion (TTE) at 45% and 75% maximal voluntary contraction (MVC) for both the flexor and extensor muscle groups of the knees, were recorded before and after three months of exercise training. All training groups showed significant decreases in body mass, BMI, total body fat, body fat percentage, WC, abdominal and visceral mass (*p* < 0.001), with a greater reduction of body mass and BMI in G75 (*p* < 0.05). Lean mass increased significantly only in G50/75 (*p* < 0.05). The insulin sensitivity and Homa-IR decreased in the three training groups (*p* < 0.01), with greater enhanced resistance in G50 compared to G75 and G50/75 (*p* < 0.05). In contrast, there were no pre-post changes in all groups for fasting blood glucose (*p* > 0.05). 1-RM and TTE of the knee flexor and extensor muscles were improved in the three groups (*p* < 0.01), with greater improvement in G50/75 for 1RM and G75 in most of the TTE parameters (*p* < 0.05). RHR decreased and 6MWD increased significantly in the three training groups (*p* < 0.01), with greater 6MWD improvement in G75 (*p* < 0.05). In conclusion, the three training intensities seem to generate benefits in terms of body composition, physiological and muscular adaptations, and insulin resistance. High training intensity resulted in greater improvements in body mass, BMI, and endurance and strength, whereas moderate training intensity resulted in greater improvements of insulin resistance and homo-IR. Following alternate-intensity training, greater improvements were observed in lean mass and maximal strength performance.

## 1. Introduction

During the last few decades, the obesity epidemic has expanded across the globe among all age groups [1]. Obesity is characterized by an accumulation of abdominal, particularly visceral fat, which is a determining factor in insulin resistance, especially in skeletal muscles [2,3]. Waist circumference (WC) is the preferred indicator of central obesity because it assesses visceral and subcutaneous abdominal fat [4]. Two key features of the pathogenesis of type 2 diabetes mellitus (T2DM) are a decrease in the ability of insulin to fulfil its normal physiological role, insulin resistance [5], and the inability of pancreatic β cells to adequately secrete insulin [6]. Insulin-resistant subjects are largely determined by an increased postabsorptive (“basal”) hepatic glucose production (HGP) and a reduced ability of insulin to suppress HGP, which contributes to hyperglycemia [7]. In these subjects, impaired hepatic self-regulation is expressed by an increased production of hepatic glucose [8]. Abnormalities in muscle metabolism play a decisive role in the development and aggravation of these pathologies [9], mainly concerning the metabolism of muscle, both in its carbohydrate and lipid components, and muscle typology and vascularization. Abnormalities in carbohydrate metabolism are directly related to insulin resistance. It is worth noting that muscles are the main target tissues for insulin action, and they are physiologically responsible for 70% of glucose metabolism and 85 to 90% of insulin-dependent metabolism [10].

In healthy individuals, the ability of insulin to stimulate glucose uptake and storage is proportional to the activation of the muscle glycogen synthetase (GS), by its dephosphorylation, under the action of GS phosphatase [11]. However, GS activity is reduced in insulin-resistant obese patients [12]. This anomaly appears earlier and seems to worsen with the progression of diabetes [12]. In some cases, the defect in muscle GS activation has been linked to decreases in GS phosphatase activity [13]. Additionally, a marked decrease in carbohydrate oxidation has been detected in insulin-resistant subjects, and in particular diabetics. This can be explained by histological and biochemical changes in the muscles of obese patients, who have a lower proportion of slow oxidative muscle fibers (type I) in favor of fast glycolytic fibers (type II) [14]. From a biochemical perspective, the decrease in the oxidative use of glucose can be explained by a decrease in oxidative enzymes, including pyruvate dehydrogenase (PDH), which is accompanied by an increase in certain glycolytic enzymes, such as phosphofructokinase-1 (PFK-I), and an increase in creatine kinase (CK) [15]. 

It is well established that physical inactivity is associated with global disease burden [16], insulin resistance and the low oxidative capacity of skeletal muscle [17]. Contrarywise, the regular practice of physical activity has been suggested to help prevent T2DM (50% reduction in the incidence of T2DM in subjects at high metabolic risk) and the management of T2DM by enhancing glycemic control [18,19,20] and reducing associated comorbidities (improvement in blood pressure and lean mass, as well as reductions in lipid profile, fat mass, and insulin resistance) [16,18,19,21]. However, the required dose of exercise (i.e., duration, frequency, and intensity), specifically the optimal exercise intensity, to maximize insulin sensitivity and β-cell function in those at risk of, or with, T2DM is still under debate. As the benefits of insulin action are rapidly reversed [22,23], previous reports recommended a longer duration of exercise (45 to 60 min) [24,25] 3–5 times per week. Regarding exercise intensity, the most common recommendations suggest low-to-moderate exercise intensity [24,25]. However, more recent evidence indicates high-intensity exercise training confers high cardiometabolic benefits (e.g., reduction in abdominal visceral fat, total cholesterol, and/or blood pressure), [26] and appears effective in improving insulin sensitivity, in people at risk of, or with, T2DM [27]. Similarly, there is growing interest and appreciation for strength training to induce several health benefits including obesity prevention, increased muscle strength, the prevention of sarcopenia, the reduction of body fat, and the preservation of bone mineral density, as well as enhanced mitochondrial oxidative capacity, glucose transport, and glycemic control [28,29,30,31]. 

To our knowledge, no studies have conducted a comprehensive comparison of the effects of combined training program (endurance-based training with strength exercise) at different exercise intensities (moderate, high, and alternated) on the biological and histological parameters affecting insulin resistance in people at risk of T2DM. 

This study aimed to compare the impact of 12 weeks of a moderate vs. high vs. alternated intensity training program, combining endurance and strength exercises, on anthropometric and body composition parameters, physiological and muscular adaptations, and insulin sensitivity among obese young women.

## 2. Materials and Methods

### 2.1. Subjects

In all, 115 obese women were screened during the pre-randomization process. Ten declined to participate because of family and work responsibilities. Fifty-seven were excluded due to a history of coronary artery disease (13 subjects), clinically significant inflammatory processes in the airways (15 subjects), abnormal renal function (8 subjects), severe metabolic syndrome (16 subjects), and hypothyroidism (5 subjects). A total of 48 women were enrolled and randomized into 4 groups (control- and three training groups; n = 12 in each group). Data of 5 subjects were eliminated from the analysis due to their poor involvement in the intervention process (<70%). Data of 43 participants (i.e., 10 from the control group (G0), 11 from the low-intensity-training group (G50), 10 from the high-intensity-training group (G75), and 12 from the alternate-intensity-training group (G50/75)) were analyzed. Detailed characteristics of each group are presented in Table 1. The three training programs were well tolerated by the involved participants, and no exercise-related accidents were recorded throughout the training period.

To be included in the study, participants were required to be between 20–30 years; have a BMI between 30 and 40 kg/m^2^ and have a waist circumference above 88 cm. The exclusion criteria were (i) secondary forms of obesity and/or secondary forms of hypertension; (ii) T2DM; (iii) coronary artery disease history; (iv) stroke (including transient ischemic attack); (v) congestive heart failure; (vi) clinically significant arrhythmias or conduction disorders; (vii) malignancy; (viii) history of use of any dietary supplements within 3 months before the study; (ix) poorly controlled hypertension (mean systolic blood pressure (SBP) > 140 mm Hg and/or mean diastolic blood pressure (DBP) > 90 mm Hg) during the month before the trial and/or necessity to modify antihypertensive treatment in the 3 months before the trial; (x) lipid disorders requiring medication either in the 3 months before the trial or during the trial; (xi) abnormal liver, kidney, or thyroid gland function; (xii) clinically significant acute or chronic inflammatory process within the respiratory, digestive, or genitourinary tract, or in the oral cavity, pharynx, or paranasal sinuses, or connective tissue disease or arthritis; (xiii) infection history in the month before the trial; (xiv) nicotine, alcohol, or drug abuse; (xv) pregnancy or childbirth at enrollment or in the 3 months before enrollment; (xvi) current lactation or lactation in the 3 months before enrollment. All participants provided a written informed consent to participate in the study. The study was conducted according to the Declaration of Helsinki and the protocol was fully approved by the local ethics committee of the Habib Bourguiba University Hospital in Sfax.

### 2.2. Experimental Design

In the present randomized controlled trial (RCT), participants were tested before and after 12 weeks of intervention. In the present RCT, subjects were first divided into 4 equal groups matched for age, BMI, and WC. Within each matched group, participants were randomly assigned to one of the experimental groups to compare outcomes. A simple random sampling technique was employed using the Excel software to generate random numbers for each subject in the sampling frame. Additionally, to prevent bias in evaluating training-intensity effects, technician blinding was employed. The three training groups (G50, G75, and G50/G75) completed 12 weeks of combined (endurance and strength exercises) training program. Regarding the endurance based-exercise, G50 underwent moderate-intensity training (brisk walking) at 50% of the heart rate reserve (HRR), G75 exercised at high-intensity-training (jogging) at 75% of HRR, and G50/75 exercised at alternate-intensity-training (brisk-walking/jogging) at 50–75% of HRR. G0 completed all tests but did not train. 

During the study, all participants were encouraged to continue their normal nutritional habits, avoid any dietary supplements, and report their dietary intake during the dietary intake interviews. Pre- and post-intervention measurements consisted of (i) anthropometric and body composition parameters including body mass, BMI, free- and total-fat masses and waist circumference (WC); (ii) physical fitness measurements including endurance capacity, evaluated as 6-min walk distance (6MWD) performance, and resting heart rate (RHR); (iii) biological parameters including fasting blood glucose, and insulin sensitivity and resistance (Homa-IR); and (iv) the voluntary maximum level of strength (i.e., maximum repetition (1-RM)), and burn-out time (i.e., time to exhaustion (TTE) at 45% and 75% maximal voluntary contraction (MVC)) for both the flexor and extensor muscle groups of the knees.

### 2.3. Anthropometric and Body Composition Measurements

Anthropometric measurements were conducted with the subjects wearing light clothing and no shoes. Weight was measured to the nearest 0.1 kg by an electronic Tefal scale and height to the nearest 0.5 cm by a wooden cloth for each subject. BMI was calculated as weight divided by height squared (kg/m^2^). Obesity was defined as BMI ≥ 30 kg/m^2^.

WC, generally used as a marker of abdominal obesity, was measured to the nearest cm. As recommended by international guidelines, WC was measured with a non-deformable tape ruler between the lower rib margin and the iliac crest, at the end of a gentle expiration). Android obesity is defined by a WC greater than 88 cm in women [32].

Body composition (i.e., free fat mass and total fat mass) and body mass were determined using bioelectrical impedance (TANITA BODY, composition analyzer mode, TBF-300). This method of measurement has been validated for use in obese women [33]. All bioimpedance measurements were taken during the same morning hours, in the same standard anatomical position, with participants dressed in standardized clothing (barefooted with sport shorts and a T-shirt). Participants were asked to refrain from eating and drinking for at least 12 h before the test, as well as from vigorous activity and consuming caffeine (e.g., coffee, energy drinks, and tea) and alcohol for at least 24 h before the test.

### 2.4. Physical Capacity Measurements

The 6MWT is considered an important tool in clinical practices. According to Morinder et al. [34] and Reschke et al. [35], the 6MWT has good reproducibility and known group validity and can be recommended for use in clinical practice, even in obese children aged 8 to 16 years. In addition, the 6MWT performed on a 15 m track is a valid field test for predicting VO2 max in healthy adults (men and women) aged 19 to 75 years, with an accuracy of approximately 1MET [36]. The 6MWT was carried out according to the protocol proposed by the American Thoracic Society [37]. Two tests were performed 20 min apart in a calibrated 30 m corridor marked by two starting and finishing blocks. The instructions were standardized, and the goal of the 6MWT was to cover as much distance as possible in six minutes. The test is performed after a rest period, and the total distance travelled is calculated at the end of the test. Only the performance with the greatest covered distance was retained.

Resting heart rate was measured following at least 5 min at rest in a sitting position using a Polar heart rate monitor (Polar Electro, S610). 

### 2.5. Biochemical Analysis

To avoid the time-of-day effect (TOD) [38,39], all blood samples were obtained between 07h00 and 08h00. Insulin, and glucose, were determined in the basal state after 12 h of fasting. The collection was carried out by a nurse at the medical sports center of the high institute of sport and physical education in Sfax. The blood collected was distributed between Fluoride + oxalate tube for glucose determination and the EDTA tube for insulin dosage. Glycaemia was measured with an enzymatic method with a 2.82% coefficient of variation (CV), with a Réactif Biomaghreb, and with a Flexor-type device. Insulin was measured with an immune-enzymatic method (CX9 Beckman coulter) with intra-assay and inter-assay CV < 10% and with a Réactif Access. Insulin resistance was assessed using the homeostatic model assessment for insulin resistance (HOMA-IR). The HOMA-IR was calculated as “fasting insulin (μUI/mL) × fasting glucose (mmol/l)/22.5” as proposed by Wallace [40].

### 2.6. Dietary and Supplement Intake

Before and after the intervention and every two weeks during the intervention, dietary intake was determined based on dietary intake interviews. The daily diets were analyzed using a computerized program (Bilnut.2.01 software package, S.C.D.A. Nutrisoft, Cerrelles, France). The intake of nutrients, total caloric intake, and caffeine consumption during the study were constant and comparable between the groups (Table 1). 

### 2.7. Evolution of 1-RM and TTE for Flexor and Extensor Knee Muscles

To assess the MVC, we used the dynamic resistance test with a maximum repetition (1-RM). 1-RM is defined as the maximum capacity or load that an individual can lift only one time. During this trial, the subject selected a weight to perform as many repetitions as possible until failure. According to Mayhew et al. [41], 1-RM prediction equations are more accurate when subjects complete < or =10 repetitions-to-failure, with the Brzycki equation being the most accurate. Therefore, for subjects who completed more than 10 repetitions to failure using the selected weight, we gradually increased the weight until reaching the one corresponding to a maximum of 10 repetitions-to-failure. To avoid power and force production reductions, at least a three-minute rest interval was used between sets, as suggested by Ammar et al. [42]. The 1-RM was then predicted using the Brzycki formula: 1RM = total weight lifted in kg/(1.0278 − (0.0278 total number of repetitions)) [43]. Due to the sedentary nature of the subjects, and to avoid injuries, each subject underwent a warmup before testing. The maximum value of 1-RM for each tested muscle group (knee extensor and flexor) was measured separately through two positions. The sitting position required for the measurement of knee extension and flexion was adjusted for each subject to ensure alignment of the knee joint to the machine’s pivot point. The maximum weight determined previously was applied to the distal and frontal end weight bar of both tibias. 

**Figure 1:**
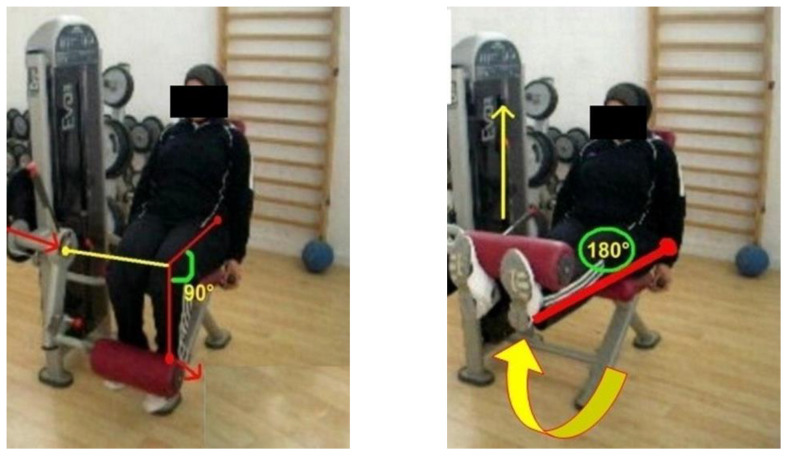
1-RM prediction of knee extensor muscles.

**Figure 2:**
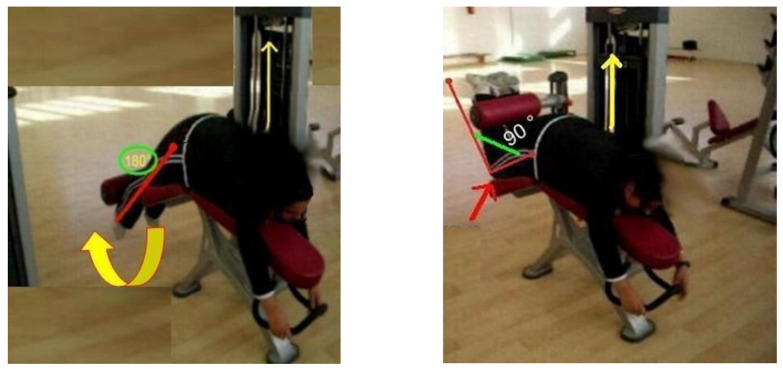
1-RM prediction of knee flexor muscles.

During the first position, the tibia moved from a normalized 90° starting position at the knee until full extension. This concentric phase was completed when the legs reached 180° and took a ground position. In the end, the tibia has taken its initial position at 90° (Figure 1). During the second position, the tibia was moved from a 180° starting position to a 90° knee flexion. When the tibia returned to its initial position of 180°, the eccentric phase was checked, and a full repeat was performed (Figure 2). All participants were given standard verbal encouragement during each MVC and visual feedback of the produced force was provided.

To avoid TOD [44] and sleep deprivation effects [45,46], all test sessions were performed in the morning by the same examiner and under the same conditions following a normal night’s sleep. Consumption of coffee and/or energy drink was prohibited during the 8 h preceding the test sessions, to avoid any ergogenic effects [47].

The use of burn-out time or TTE is increasingly common in the evaluation of physical intensity [48]. In this study, TTE corresponds to the maintenance time at 45% and 75% MVC for both the flexor and extensor muscle groups of the knees.

For the TTE measurement of the knee extensors, the predetermined load was located at the distal and frontal extremities of the two tibias. The stopwatch was activated when the two legs reach the 180° position and remain parallel to the ground. The subject was asked to keep the 180° position for as long as possible until exhaustion by the muscle endurance task used by Bigard et al. [49] and Brown and Bray [50].

For the TTE measurement of the knee flexors, the predetermined load was placed at the distal and posterior extremities of the two tibias. The stopwatch was activated when the two legs are flexed. The subject was asked to maintain the 90° position for as long as possible.

### 2.8. Training Program

Participants from G50, G75 and G50/75 participated in a 12-week-combined training program with a frequency of 5 sessions/week. A total of 60 training sessions were conducted for each group under the supervision of a qualified and certified physical education teacher and medical supervisor. Due to their initial sedentary state, participants performed a progressive load training-program. The training sessions progressed throughout the 12 weeks of training from 20 to 60 min of effective work as previously described by Mezghanni et al. [51,52]. During exercise, HR was controlled with a Polar heart rate monitor (Polar Electro, S610). The exercise intensity was adjusted on an individual basis to ensure each subject exercised at their prescribed intensity based on their target heart rate (HR) calculated from Karvonen’s method [53]. This method is based on the maximal HRR: RHR + 0.50 [(HRmax − RHR) for (50% of HRR) and RHR + 0.75 [(HRmax − RHR) for (75% of HRR). HRmax was predicted via the following formula: Theoretical HRmax = 220-age (bpm).

The exercise duration for all three training groups was the same. Exercise duration progressed from 20 to 25 min during the first and second week, to 40 min by the end of week 7, and 55–60 min by the end of week 12. As suggested by Kay et al. [54] and Mezghani et al. [51], exercise intensity was rated as moderate for G50 (walking at 50% HRR), high for G75 (jogging at 75% HRR), and alternated between moderate and high for walking/jogging for G50/75 (50% and 75% HRR). 

During the first two weeks, all groups performed 2 sets of brisk walks at an intensity of 40% of the HRR, with 2 repetitions of 5 min per set. During the remaining period, each group trained at the selected intensity (50% HRR for G50, 75% HRR for G75, and alternating 50/75% HRR for G50/75). Subjects performed 1 to 3 sets of brisk walking (G50), jogging (G75), or alternating walking/jogging (G50/75), with 1 to 2 repetitions of 7–20 min per set. During this period (weeks 3–12), the duration of the exercise (7–20 min), and the number of sets (1–3) and repetitions (1–2) varied between sessions to prevent participants’ boredom. However, the target total exercise duration per session (40 min for weeks 3–7 and 55–60 min for weeks 8–12) was respected. During the entirety of the training program, repetitions were separated by 2 min of breathing exercises, and sets were interspersed with 10 min of stretching and relaxation.

During the third training session of each week, in addition to walking or jogging, each group performed 15 min of additional circuit style training (abdominal exercises, back exercises, and squats). These exercises consisted of 3 sets of 10 repetitions for the first three weeks of the program. Thereafter, the number of repetitions increased to 20 from the weeks 4 to 6 and to 30 for the remaining period (weeks 7 to 12). 30 s recovery was taken between sets, during which the subjects performed breathing exercises. All exercises were performed with a 3 kg medicine ball.

### 2.9. Statistical Analysis

All data were analyzed using the SPSS statistical software version 11 and the statistical significance was set at *p* ≤ 0.05. The normality of the distribution was verified with the Shapiro–Wilk W-test. Results were presented as Mean ± standard deviation (SD) in tables and text. Differences among the three groups were examined using a one-way ANOVA, and Scheffe’s post hoc test was performed when these results were significant. Paired *t*-tests were used to assess changes within groups for all variables, and Bonferonni adjustments were used to interpret all t-test results.

## 3. Results

Baseline characteristics recorded before the 12-week intervention for all groups are shown in Table 1. There were no differences among groups for any of these variables. Attendance at exercise sessions did not differ significantly between exercise groups (G50 = 95.4%, G75 = 96.1%, and G50/75 = 96.5%). Analysis of the “daily dietary intake” records showed relative caloric intake did not differ between groups throughout the training period (Table 1).

Table 2 shows the change in participants’ dietary intake and anthropometric and body composition characteristics after the 12-week intervention. All training groups had a significant decrease in body mass, BMI, total body fat, body fat percentage, WC, and abdominal and visceral mass (*p* < 0.01) with a greater reduction of weight, total fat mass and WC in G75 (*p* < 0.05). There are no pre-post changes in all groups for dietary intake (*p* > 0.05).

As shown in Table 3, the three training interventions led to significant decreases in Homa-IR and insulin levels (*p* < 0.01), with the greater improvement of insulin resistance in G50 compared to G75 and G50/75 (*p* < 0.05). 

Table 4 shows the change in participants’ physical fitness. Compared to baseline measurements, the covered distance in the 6MWD test increased significantly following the three training programs (*p* < 0.01), with significantly higher improvement in G75 (29.1%), compared to G50 (19.4%) and G50/75 (20.6%) (*p* < 0.05). RHR significantly declined after the training period in all three training groups (*p* < 0.01) with a % change of −17.4%, −19.8%, and −18.7% for G50, G75, and G50/75, respectively. There were no significant differences between groups and no significant changes pre-post for G0.

In addition, the three training interventions generated significant increases in 1-RM and TTE at 45% and 75% MVC for knee flexors and extensors (*p* < 0.05, Table 5) and in 1-RM and average TTE at 45% and 75% MVC (Table 6). At post-intervention, significantly higher values in most of the 1-RM and M1RM variables were observed for G50/75 and in most of TTE variables were registered for G75 (*p* < 0.05) compared to the other groups.

## 4. Discussion

This study evaluated the effects of 12 weeks of three different training intensities, without caloric restrictions, on several anthropometric, biological parameters, as well as their impact on the control of insulin resistance and the histological changes of muscle fibers in obese women. 

Our results revealed 3 months of individualized intensity endurance training combined with strength exercise improved insulin sensitivity expressed by a significant reduction in insulinemia and HOMA-IR index. These results confirm previous findings demonstrating the crucial role of exercise-induced weight loss in the treatment of insulin resistance with well-defined intensity, dosage, and frequency [55,56]. The reduction of the HOMA-IR index in the present study may be due to the significant reduction of WC and total fat mass as well as the consequence of preserving or increasing lean body mass among the training groups. These results remain consistent, with subsequent studies indicating that WC was the indicator most strongly associated with the absolute amount of intra-abdominal or visceral fat, the fat deposit that carries the greatest risk for the establishment of insulin resistance and several other metabolic and cardiovascular complications [57,58,59]. WC is considered an index of central obesity recommended by the WHO, National Institutes of Health, the International Diabetes Foundation, and the American Heart Association for screening for risk of metabolic and cardiovascular disease [2]. 

Furthermore, in the present study, the improvement in insulin resistance was more pronounced in the low-intensity group G50 than in the high- and alternated- intensities groups. Previous studies have demonstrated that low-intensity endurance exercise is more effective than intense exercise in improving insulin resistance in people with mild insulin resistance [60,61]. In contrast, other findings indicate insulin resistance remains unaffected after 6 months of low-intensity training, while 6 months of high-intensity training reduces insulin resistance by 30% [62]. Other researchers have shown that, regardless of the exercise’s intensity, the exercise duration during training sessions has a crucial role in improving insulin sensitivity [63]. The present results further confirm that low-intensity aerobic training is associated with the greatest improvement in the insulin resistance.

In our study, the three intensities of the combined endurance and strength training programs showed significant decreases in body weight, total body fat, body fat percentage, and WC indicative of decreased abdominal and visceral fat favorable for insulin sensitivity [64,65,66]. These findings are consistent with: (i) previous research showing regular aerobic-based exercise is associated with marked reductions in body weight and fat mass in overweight and obese men and women [31,56,67,68]; and (ii) recent findings from a meta-analysis of 114 studies in obese and non-obese subjects demonstrating a significant reduction in body weight, total body fat, and body fat percentage following combined aerobic and resistance and aerobic exercise [69]. According to this meta-analysis, combined aerobic and resistance exercise training was the most effective intervention for reducing visceral adipose tissue (VAT) and subcutaneous abdominal adipose tissue (SAT) [69]. Similarly, the reduction in WC combined with a decrease in body mass index in our trained subjects suggests exercise-induced weight loss is preferentially associated with a decrease in abdominal obesity [56,70]. These results are aligned with the findings of a recent meta-analysis of 89 studies indicating significant decreases in SAT after different types of exercise [71]. In fact, exercise increases the secretion of lipolytic hormones, such as growth hormone, which activate lipolysis in adipose tissue like subcutaneous abdominal adipose tissue (SAT) [71,72]. The current reduction in abdominal obesity after the three training programs may be a key to the improvement in insulin resistance. It has been suggested SAT accounts for ∼80% of abdominal fat and is the major supplier of free fatty acids to the liver [73,74]. Therefore, a decrease in SAT may prevent hepatic steatosis and subsequently insulin resistance [75].

In addition, our results revealed greater significant reductions in body mass and BMI and a slightly greater reduction of fat mass and WC in the high-intensity training group compared to the low- and alternated-intensity groups. These results are consistent with several studies reporting a higher reduction of body mass and BMI following high- compared to low-intensity training programs [68,76,77]. Additionally, it has been demonstrated that the contribution of lipids to energy metabolism depends on the intensity and duration of exercise [78]. While low-intensity exercise mostly focuses on the oxidation of lipids as an energy source, high-intensity exercise necessitates the catabolism of glucose [79]. However, lipid oxidation, sometimes referred to as post-exercise lipolysis, is higher 24 to 48 h after high intensity exercise than after moderate intensity exercise [80]. These findings may be explained by high-intensity training inducing a profound effect on fat metabolism via lipolysis and lipid uptake by muscular tissues. Similar beneficial changes are achieved through decreasing insulinemia [81], maintaining the adrenergic receptor sensitivity [82] and/or stimulating the sympathetic nervous system [83], and/or activating the oxidation of the fatty acids via mitochondrion [40]. Specific adaptations to high intensity exercise have been shown to include 10.2-fold duplication for PGC-1α mRNA (favoriting greater expression of PGC-1 α) and increased mitochondrial protein synthesis [84]. Both adaptations have previously been suggested to promote the expression of oxidative enzymes in muscle [85] and producing consequently a greater lipid oxidation [80]. 

At the same time, our results demonstrated a variation in fat free mass from pre- to post-intervention with negligible change in G75 (−0.4%), a non-significant increase of 1.8% in G50 and a significant increase of 2.1% in G50/75. Considering muscles are important for glucose storage and may help reduce visceral fat, we postulate that the present increase in lean body mass, specifically for G50 and G50/75, can be explained (in part) by the greater reduction of insulin resistance following the low-intensity based-training (particularly in G50), compared to the high-intensity training program (G75). This assumption is in line with previous reports indicating that, following an aerobic training program, increases in mitochondrial content and glucose transporters (Glut4) may be the origin of improved insulin sensitivity [86,87]. In the same context, Schrager et al. [88], and Akhmedov and Berdeaux [89], reported that improvements in lean body mass promoted a reduction in pro-inflammatory cytokines, particularly interleukin-6 and tumor necrosis factor-alpha, implicated as an important factor in obesity-associated insulin resistance and the pathogenesis of type 2 diabetes [90].

Concerning the maximum voluntary strength, our findings demonstrate an amelioration of 1-RM and M1RM following the three training programs, which is consistent with the findings of Moliner-Urdiales et al. [91] in adolescents. As the development of strength was accompanied by an increase in lean mass for G50 and G50/75, the present increase in MVC-related parameters in both groups can be attributed to either an increase in muscle mass through the process of hypertrophy or muscle fiber hyperplasia. Particularly G50/75, which alternated high and low-intensity exercises, showed the highest change in lean mass, and had the greatest improvement in the 1-RM knee extension and average 1-RM for both knee muscle groups (extensors and flexors). The significant development in maximal voluntary strength in G75, which was not accompanied with increased lean mass, confirms previous suggestions that strength improvement can occur outside of any structural changes in the muscle, independently through neural adaptations [92]. This has been seen previously in females who previously demonstrated strength increases by 20% to 40%, without any increase in lean mass [93]. Similarly, the improvement in insulin sensitivity and resistance in G75 may be due, in part, to possible neural improvements in this group, as a high correlation has been reported between diabetes mellitus obesity and peripheral neuropathy [94]; and high-intensity exercise training recently showed a beneficial effect on neuronal functions [95,96]. Concerning G50 and G50/75, the improvement in insulin sensitivity and resistance, which was accompanied by increased lean mass, may be explained by a raise in slow and oxidative slow muscle fibers (type I and II). Lillioja et al. [14] reported a significant inverse correlation between the percentage of these fiber types and the degree of adiposity and/or the degree of insulin resistance. 

We also found an improvement in the TTE at 45% and 75% RM following the three training programs. We suggest this enhancement is attributed to various physiological and training adaptations, such as the enhancement of muscle energy reserves, carbohydrate and lipid intakes, vascular bed growth at the muscle level (ensuring better oxygenation), and better use of energy substrates [97,98]. The greater burn-out time registered for the majority of TTE tests in G75 compared to the remaining training group suggests high-intensity exercise programs generate the optimal muscle histological adaptation favorable for enhanced muscle contraction maintenance, despite the absence of lean mass improvement in this group. 

Our results indicate the three training programs had a beneficial impact on aerobic capacity, as evidenced by a significant increase in 6MWD and a decrease in RHR. Previous reports reveal physical exercise interventions improve endurance performance [99,100], BMI, abdominal and visceral adiposities, insulin resistance, and metabolic profiles [101], with a negative correlation between the changes in 6MWD and WC (a marker of visceral adipose tissue) [102]. Our findings are consistent with the above and showed the greater improvement in the 6MWD for the high-intensity physical activity group (G75), and was accompanied by significantly greater reduction in body mass and BMI and a slightly greater reduction in fat mass and WC compared to G50 and G50/75. Additionally, all trained subjects decreased their RHR, which consequently reflects an improvement in their level of physical fitness and cardiovascular health [103]. Our results are in line with studies demonstrating that, after 4 to 6 weeks of adjusted training, bodily systems can adapt by increasing mitochondrial density and perfusion as well as increasing oxygen availability for the Krebs cycle or glycolytic cycle [104], leading to a reduction in resting heart rate and blood pressure [105]. 

### Study Strengths and Limitations

Subjects’ compliance in all investigated groups was over 85% (i.e., ≈92% for G0 and G75, ≈85% for G75, and 100% for G50/75), which enhances the study findings. A large variety of parameters were measured and compared in the same study groups. The inclusion and exclusion criteria were very strict, which allowed the enrollment of a homogenous group of subjects who were free of illnesses and eliminates the influence of disrupting factors. The major limitation of this study is the relatively small number of subjects completing the intervention, likely resultant of the strict inclusion and exclusion criteria.

## 5. Conclusions

In conclusion, moderate, high, or alternated training intensity appeared to provide benefits in terms of body composition, physiological and muscular adaptations, and insulin resistance with greater improvements in: (i) body mass, BMI, endurance and strength capacities following high-training intensity; (ii) insulin resistance and homo-IR following moderate training-intensity; and (iii) lean mass and maximal strength performance following alternated-intensity. These findings may have implications for the practical recommendation of simple and low-cost management programs for women suffering from abdominal obesity. Such programs should also consider the clinical and physical condition of the subjects, as well as their preference for walking or jogging.

## Figures and Tables

**Table 1:** Baseline characteristics of the study population.

Variables	Groups				Difference between Groups	
	G0 (n = 10)	G50 (n = 11)	G75 (n = 10)	G50/75 (n = 12)		
Age (years)	25 ± 4	27 ± 4	25 ± 5	28 ± 5	NS	
Anthropometric Measurements						
Body mass index (kg/m^2^)	33.7 ± 1.8	34.1 ± 3.5	32.9 ± 1.8	32 ± 2.1	NS	
Body mass (kg)	85.8 ± 9.6	94.3 ± 10.5	86.7 ± 10.5	82.4 ± 5.7	NS	
Waist circumference (cm)	100.7 ± 9.8	106.4 ± 7.4	100.1 ± 5.3	101.3 ± 7.1	NS	
Physical Capacity Measurement						
Walk distance (m)	535.5 ± 51.4	494.6 ± 26.3	567.7 ± 51.3	566.8 ± 56.3	NS	
Heart rate (bat/min)	82.5 ± 5.8	77 ± 4.6	77.1 ± 5.6	80.7 ± 9.1	NS	
Calorie intake (kcal/day)	2809 ± 571	2576 ± 566	2894 ± 682	2889 ± 760	NS	

G0: control group; G50: Low-intensity training group; G75: High-intensity training group; G50/75: Alternate low and high-intensity group; NS: no significant difference before the intervention period.

**Table 2:** Change in energy intake. anthropometric and body composition measurements following the 12-week intervention.

Characteristic	Groups				Pre vs. Post			
	G0	G50	G75	G50/75	G0	G50	G75	G50/75
Δ Energy intake (kcal/day)	−39 ± 159	−212 ± 323	−229 ± 554	−279 ± 941	NS	NS	NS	NS
Δ BMI (kg/m2)	−0.2 ± 0.7	−1.2 ± 0.8 *	−2.4 ± 1.4 *#	−1.2 ± 1.1 *+	NS	†	†	†
Δ Body mass (kg)	−0.5 ± 1.7	−3.1 ± 2 *	−5.3 ± 2.2 *#	−2.4 ± 2.3 *+	NS	†	†	†
Δ Fat Mass (Kg)	−0.1 ± 1.2	−3.9 ± 2.5 *	−5.1 ± 1.7 *	−3.5 ± 2.5 *	NS	†	†	†
Δ % Fat Mass	0.1 ± 0.7	−2.8 ± 2.2 *	−3.7 ± 2.1 *	−3.2 ± 2.4 *	NS	†	†	†
Δ Fat free mass (Kg)	−0.3 ± 0.5	1 ± 2.1	−0.2 ± 1.6	1 ± 1	NS	NS	NS	†
Δ WC (cm)	−0.3 ± 1.6	−8.6 ± 3 *	−9.5 ± 2.6 *	−7.5 ± 5 *	NS	†	†	†
Δ Waist Hip (cm)	−0.4 ± 0.6	−5.2 ± 2.6 *	−6.6 ± 3.1 *	−5.7 ± 4 *	NS	†	†	†
Δ Waist-Hip Ratio (cm)	0 ± 0	0 ± 0	0 ± 0	0 ± 0	NS	†	†	†

†: Significant difference in comparison with pre-intervention at *p* < 0.01. *: Significant difference in comparison with G0 at *p* < 0.05. #: Significant difference in comparison with G50 at *p* < 0.05. +: Significant difference in comparison with G75 at *p* < 0.05.

**Table 3:** Change in biochemical measurements following the 12-week intervention.

		Groups				Pre vs. Post			
		G0	G50	G75	G50/75	G0	G50	G75	G50/75
Glucose (mmol/L)	Baseline	4.9 ± 0.6	5 ± 0.7	4.6 ± 0.3	5 ± 0.4	NS	NS	NS	NS
	After intervention	4.9 ± 0.7	4.6 ± 0.5	4.5 ± 0.5	5.2 ± 0.4				
	Change from pre to post	−0.1 ± 0.3	−0.4 ± 0.7	−0.2 ± 0.7	0.2 ± 0.5				
Insulin (µUI/L)	Baseline	11 ± 6.2	10.4 ± 4.7	8 ± 5.1	7.2 ± 3.7	NS	†	†	†
	After intervention	11.2 ± 6.3	6.6 ± 3.2	5.7 ± 3.2	4 ± 2				
	Change from pre to post	0.2 ± 0.6	−3.8 ± 4 *	−2.3 ± 2.7	−3.2 ± 3.8 *				
HOMA-IR	Baseline	2.5 ± 1.5	2.3 ± 1.1	1.6 ± 1.1	1.6 ± 0.9	NS	†	†	†
	After intervention	2.5 ± 1.7	0.7 ± 0.7	1.1 ± 0.6	0.9 ± 0.5				
	Change from pre to post	0 ± 0.2	−1.6 ± 0.8 *	−0.5 ± 0.6 #	−0.7 ± 0.9 *#				

†: Significant difference in comparison with pre-intervention at *p* < 0.01. *: Significant difference in comparison with G0 at *p* < 0.05. #: Significant difference in comparison with G50 at *p* < 0.05.

**Table 4:** Change in physical capacity measurements following the 12-week intervention.

Variables	Groups				Pre vs. Post			
	G0	G50	G75	G50/75	G0	G50	G75	G50/75
WD (m)	4.1 ± 8.6	96.1 ± 25.1 *+	165.2 ± 28.9 *	117 ± 61.2 *+	NS	†	†	†
HR (bat/min)	−0.1 ± 1.3	−13.4 ± 4 *	−15.3 ± 5.9 *	−15.1 ± 10.2 *	NS	†	†	†

WD: walk distance. HR: heart rate. †: Significant difference in comparison with pre-intervention at *p* < 0.01. *: Significant difference in comparison with G0 at *p* < 0.05. +: Significant difference in comparison with G75 at *p* < 0.05.

**Table 5:** Variation in 1-RM and time to exhaustion (TTE) at 45% and 75% RM for the knee flexors and extensors.

Variables	Groups				Pre vs. Post			
	G0	G50	G75	G50/75	G0	G50	G75	G50/75
1-RM knees extensors (Kg)	−0.49 ± 1.42	9.73 ± 7.72 *	14.69 ± 14.58 *	30.02 ± 10.59 *#+	NS	†	†	†
1-RM knees flexors (Kg)	−0.42 ± 0.81	9.15 ± 6.27 *	6.96 ± 5.41 *	11.14 ± 6.33 *	NS	†	†	†
Extensors TTE at 45% RM (sec)	−4.3 ± 12.3	109.8 ± 30.9 *	164.3 ± 52.5 *#	85.5 ± 30.1 *#+	NS	†	†	†
Extensor TTE at 75% RM (sec)	−1.2 ± 4.3	66.2 ± 24.5 *	89.4 ± 30.1 *#	46.3 ± 9.5 *#+	NS	†	†	†
Flexor TTE at 45% RM (sec)	−3.4 ± 4.5	66.2 ± 28.3 *	66.4 ± 21.3 *	48 ± 25 *	NS	†	†	†
Flexor TTE at 75% RM (sec)	−1.2 ± 4.3	32.8 ± 15.9 *	39 ± 24.3 *	34.5 ± 10.1 *	NS	†	†	†

†: Significant difference in comparison with pre-intervention at *p* < 0.01. *: Significant difference in comparison with G0 at *p* < 0.05. #: Significant difference in comparison with G50 at *p* < 0.05. +: Significant difference in comparison with G75 at *p* < 0.05.

**Table 6:** Variation in average 1RM (M1RM) and average time to exhaustion (TTE) at 45 and 75% RM.

Variables	Groups				Pre vs. Post			
	G0	G50	G75	G50/75	G0	G50	G75	G50/75
M1RM Groupes (Kg)	−0.45 ± 0.7	9.44 ± 6.61 *	10.83 ± 6.53 *	20.58 ± 6.62 *#+	NS	†	†	†
Average TTE at 45% RM (sec)	−3.9 ± 7.2	88 ± 26.1 *	115.4 ± 30.5 *#	66.8 ± 20.7 *#+	NS	†	†	†
Average TTE at 75% RM (sec)	−1.2 ± 3.4	49.5 ± 13.5 *	64.2 ± 19.7 *#	40.4 ± 7.2 *+	NS	†	†	†

†: Significant difference in comparison with pre-intervention at *p* < 0.01. *: Significant difference in comparison with G0 at *p* < 0.05. #: Significant difference in comparison with G50 at *p* < 0.05. +: Significant difference in comparison with G75 at *p* < 0.05.

## Data Availability

Data are available from the first author upon reasonable request.
